# Complete Genome Sequence of Bacillus cereus Strain CPT56D-587-MTF, Isolated from a Nitrate- and Metal-Contaminated Subsurface Environment

**DOI:** 10.1128/mra.00145-22

**Published:** 2022-04-27

**Authors:** Jennifer L. Goff, Lauren M. Lui, Torben N. Nielsen, Michael P. Thorgersen, Elizabeth G. Szink, John-Marc Chandonia, Farris L. Poole, Jizhong Zhou, Terry C. Hazen, Adam P. Arkin, Michael W. W. Adams

**Affiliations:** a Department of Biochemistry and Molecular Biology, University of Georgia, Athens, Georgia, USA; b Environmental Genomics and Systems Biology Division, Lawrence Berkeley National Laboratory, Berkeley, California, USA; c Institute for Environmental Genomics, University of Oklahoma, Norman, Oklahoma, USA; d Department of Microbiology and Plant Biology, University of Oklahoma, Norman, Oklahoma, USA; e Oak Ridge National Lab, Oak Ridge, Tennessee, USA; f Department of Civil and Environmental Engineering, University of Tennessee, Knoxville, Tennessee, USA; g Department of Bioengineering, University of California, Berkeley, Berkeley, California, USA; University of Rochester School of Medicine and Dentistry

## Abstract

Bacillus cereus strain CPT56D-587-MTF was isolated from nitrate- and toxic metal-contaminated subsurface sediment at the Oak Ridge Reservation (ORR) (Oak Ridge, TN, USA). Here, we report the complete genome sequence of this strain to provide genomic insight into its strategies for survival at this mixed-waste site.

## ANNOUNCEMENT

Strain CPT56D-587-MTF was isolated from subsurface sediment (long −84.27335°, lat 35.977268°, depth 535.94 cm) from the Oak Ridge Reservation (Oak Ridge, TN, USA), which is contaminated with legacy uranium and nitrate waste (1). Sediment (1 g) was inoculated into anoxic modified Reasoner’s 2A (R2A) medium (2) (10 mM nitrite, 100 mM KH_2_PO_4_, pH 5.5). Following room temperature incubation for a week, strain CPT56D-587-MTF was recovered by streak-plating onto LB agar. A complete genome sequence was obtained to provide insight into its survival strategies. CPT56D-587-MTF was grown for 24 h in R2A medium (30°C, 200 rpm). The cell pellet was digested by resuspension in 750 μL phosphate-buffered saline (PBS) and incubated at 37°C for 30 min with 25 μL MetaPolyzyme (Sigma-Aldrich) and 25 μL lytic enzyme solution (Qiagen), followed by digestion in 167 μL 6× buffer B1 (300 mM Tris-Cl [pH 8.0], 300 mM EDTA [pH 8.0], 3% Tween 20, 3% Triton X-100) (Qiagen), 35 μL proteinase K, and 2 μL RNase A with incubation at 50°C and 50 rpm for 30 min. The lysate was processed using the Genomic-tip 20/G kit (Qiagen) per the manufacturer’s directions.

High-molecular-weight (HMW) DNA was prepped for Nanopore sequencing. End repair was performed using the NEBNext companion module for Oxford Nanopore Technologies ligation sequencing (New England BioLabs) according to the manufacturer’s instructions. The native barcoding expansion (EXP-NBD104; Oxford Nanopore Technologies) and ligation sequencing (LSK-SQK109; Oxford Nanopore Technologies) kits were used for barcoding and adapter ligation, respectively. The library was sequenced on a R9.4.1 flow cell on a MinION device (Oxford Nanopore Technologies). HMW DNA was prepped for Illumina library creation by needle shearing. The DNA was not size selected. The Illumina library was generated using the Illumina DNA prep kit (catalog number 20018705; previously, Nextera) with indices from set A primers (catalog number 20027213) according to the manufacturer’s instructions and sequenced using 2 × 150-bp reads on a NovaSeq 6000 instrument by Novogene.

The sequencing read data were quality filtered and trimmed before assembly. For the Illumina data, adapters were removed in-house by Novogene. After quality filtering, there were 3,246,490 Illumina reads. These data were processed using BBTools v38.86. The processing was done in two passes (3): (i) bbduk.sh was run (*ktrim = r k = 23 mink = 11 hdist = 1 ref = adapters*.*fa tbo tpe 2*) to remove the remaining Illumina adapters given in adapters.fa (standard Illumina adapters); (ii) for quality filtering and trimming and to remove Illumina PhiX174 spike-ins given in the file phix174 Illumina.fa, bbduk.sh was run again (*bf1 k = 27 hdist = 1 qtrim = rl trimq = 17 cardinality = t ref = phix174_Illumina*.*fa*). The Nanopore sequencing yielded 136,213 raw reads (*N*_50_/*N*_90_, 11,659/2,738 bp). Nanopore base calling, adapter removal, demultiplexing, and quality filtering were performed using Guppy v4.0. Assembly was performed with the Nanopore and Illumina reads using the hybrid assembler Unicycler v0.4.8 (4) (default parameters). The Unicycler logs were checked to confirm that the assembly passed the quality thresholds and that the DNA elements were circularized. This is indicated in the “Rotating completed replicon” section of the Unicycler log. Only contig 6 was not circular.

The completed genome contains 6,548,342 bp in 9 contigs (G+C content, 35.37%). Contig 1 is the circularized chromosome, and contigs 2 to 9 are putative plasmids (Table 1). Genome annotation was performed using RASTtk v1.073 (5) in the DOE Systems Biology Knowledgebase (KBase) with default parameters (https://kbase.us/n/105874/55/) (6, 7). Taxonomic assignment performed using GTDB-Tk-v1.7.0 (8) in KBase (default parameters) identified the strain as a Bacillus cereus species (GenBank accession number GCA_000007825.1), with an average nucleotide identity of 98.58%. Finally, the assembled genome was deposited in GenBank and reannotated using the NCBI Prokaryotic Genome Annotation Pipeline v5.3 (9).

**TABLE 1:** Contig assembly information

Contig	Length (bp)	Circularity	Coverage (×)	GenBank accession no.
1	5,668,734	Circular	112	CP090081.1
2	448,451	Circular	269	CP090082.1
3	99,643	Circular	260	CP090083.1
4	83,506	Circular	312	CP090084.1
5	83,392	Circular	236	CP090085.1
6	72,739	Linear	310	CP090086.1
7	70,091	Circular	250	CP090087.1
8	15,910	Circular	3,740	CP090088.1
9	5,876	Circular	1,242	CP090089.1

### Data availability.

This whole-genome sequencing project has been deposited at GenBank under the accession number GCA_021391515.1. The raw sequence reads have been deposited in the SRA under the accession numbers SRR17696030 (Illumina short reads) and SRR17696029 (Oxford Nanopore long reads). All KBase analyses are publicly available in the KBase static narrative (https://kbase.us/n/105874/55/).
